# Nutritional Knowledge of UK Coaches

**DOI:** 10.3390/nu6041442

**Published:** 2014-04-10

**Authors:** Emma Cockburn, Alistair Fortune, Marc Briggs, Penny Rumbold

**Affiliations:** 1 London Sport Institute, Middlesex University, London NW4 4BT, UK; 2 Department of Sport, Exercise and Rehabilitation, Faculty of Health and Life Sciences, Northumbria University, Tyne and Wear NE1 8ST, UK; E-Mails: alifortune@gmail.com (A.F.); marc.a.briggs@northumbria.ac.uk (M.B.); penny.rumbold@northumbria.ac.uk (P.R.)

**Keywords:** sport nutrition knowledge, nutrition training, coaching

## Abstract

Athletes obtain nutritional information from their coaches, yet their competency in this area is lacking. Currently, no research exists in the UK which has a different coach education system to many other countries. Therefore, the aim of this study was to evaluate the sports nutrition knowledge of UK coaching certificate (UKCC) level 2 and 3, hockey and netball qualified coaches. All coaches (*n* = 163) completed a sports nutrition questionnaire to identify: (a) if they provided nutritional advice; (b) their level of sport nutrition knowledge; and (c) factors that may have contributed to their level of knowledge. Over half the coaches provided advice to their athletes (*n* = 93, 57.1%), even though they were not competent to do so. Coaches responded correctly to 60.3 ± 10.5% of all knowledge questions with no differences between those providing advice and those who did not (*p* > 0.05). Those coaches who had undertaken formal nutrition training achieved higher scores than those who had not (*p* < 0.05). In conclusion, UK sports coaches would benefit from continued professional development in sports nutrition to enhance their coaching practice.

## 1. Introduction

Optimising nutritional intake has been demonstrated to elicit peak performance levels [1,2,3] from subsequent enhancement of recovery processes, body mass control, effective hydration, reductions in illness and injury, coinciding with increased confidence as a result of a more prepared mental state for competition [4]. Irrespective of the competition level and type of sport prolonged energy deficit may lead to decreased performance and health, including iron deficiency anemia and immunosuppression [5]. Special consideration should also be noted for adolescent athletes, as adequate energy intake is essential to maintain a number of factors such as growth, health, body mass and maturation [6]. Furthermore, there are many detrimental effects of chronic inadequate energy intake in adolescents such as short stature, delayed puberty, poor bone health and increased risk of injury [6].

Athletes have misconceptions about optimal nutrition and energy requirements, relying on a variety of resources to inform practice. Athletes have sought advice from strength and conditioning coaches, dieticians, peers, family, media and independent research [1,7,8,9]. However coaches have been identified as the predominant source. Research investigating the nutritional knowledge of athletes in a range of sports globally provides supporting evidence that understanding of optimal strategies is inadequate [4,7,10,11,12].

Sport coaches are integral to the progression and development of athletes, imparting knowledge and providing decision-making contexts via the coach-athlete relationship [13]. Coaches are faced with the inevitable challenge of both advocating nutritional guidance and monitoring nutritional practices of athletes, with evidence highlighting the issue that many athletes are within a negative energy balance due to insufficient consumption of nutrients to sustain training and performance requirements [5]. Research identifies that from the vast array of information sources available to the coach the most common resource used to gain nutritional information was magazine articles [14,15]. Recommendations within such magazine articles contain unregulated sources of information lacking research-informed evidence, with questionable reliability and scientific rigour. In contrast a small number of coaches were found to improve nutritional understanding, when referring to more credible sources such as registered dieticians and nutritionists [1,16]. However, albeit within its infancy, the relatively limited research from North and South America, and New Zealand investigating coaches’ nutritional knowledge [1,3,14,15] provides unequivocal evidence that the majority of coaches possess inadequate levels of nutritional knowledge. Furthermore, findings conclude that coaches are not suitably informed to impart nutritional recommendations and strategies to athletes [1,3,14,15].

With an increasing amount of research providing evidence of coaches’ lack of correct nutritional knowledge there is a rising concern regarding the dissemination of factually incorrect and unsubstantiated information to athletes. Conclusions drawn from relevant research indicate a need for additional nutrition training [1,3,14,15,16] whilst also ensuring coaches are acquiring knowledge from reliable sources. In an ideal situation, athletes would have access to an accredited sport nutritionist; however, with the exception of top elite athletes this is not feasible, presenting an extremely challenging solution. A more realistic alternative is having appropriately trained coaches who can influence athletes’ energy intake directly.

With the heavy reliance on coaches to provide nutritional information it is vital to understand the level of knowledge to provide an insight in to the appropriateness of recommended nutritional strategies given to UK athletes. Currently no research investigating coaches’ nutritional knowledge exists in the UK, which provides a different coach education system to many other countries. Therefore, the aim of this study was to evaluate the sports nutrition knowledge of UK coaching certificate (UKCC) level 2 and 3 qualified coaches.

## 2. Experimental Section

### 2.1. Participants

One hundred and sixty three UKCC level 2 (*n =* 136) and level 3 (*n =* 27) hockey and netball coaches participated in the study. Initially 213 coaches responded to emails sent to all netball and hockey coaches registered with their National Governing Body, however, due to incomplete surveys their responses were removed from the data analysis. The sample was made up of 105 netball coaches and 58 hockey coaches. There was a relatively equal spread of ages across the ranges (18–30 years = 48; 31–40 years = 28; 41–50 years = 48; over 51 years = 39). Over half of the sample (68.7%) were qualified at undergraduate level or above (masters or PhD).

### 2.2. Procedure

Following Institutional ethical approval the questionnaire was distributed to potential participants via an email with a link to SurveyMonkey^®^. Emails containing the SurveyMonkey^®^ URL were distributed by a contact at the relevant National Governing Body to all coaches registered on their database. The use of an on-line survey program allowed tracking and managing of the large data set.

### 2.3. Sports Nutrition Knowledge Questionnaire

A previously psychometrically validated and reliable sports nutrition questionnaire [17] was distributed to the potential participants. This questionnaire has been previously used to evaluate the sports nutrition knowledge of club rugby coaches in New Zealand [14]. Terminology used within the questionnaire was updated to ensure it was appropriate for a UK audience and those working within any team sport.

The questionnaire contained two main sections. The first section was used to evaluate the demographics of the coaches including age, length of time coaching, previous playing experience, qualifications, nutrition training, rating of their own nutrition knowledge and whether they imparted advice to their athletes.

The second part of the questionnaire, which had been validated, was comprised of 88 sports nutrition knowledge questions divided into five sub-categories; nutrients (42 questions), fluids (9 questions), recovery (11 questions), weight control (15 questions), and supplements (11 questions). Each question could be answered “yes”, “no” or “unsure”, to discourage coaches from guessing at answers and allow differentiation between those possessing accurate knowledge, incorrect knowledge, and those who did not have any knowledge [14].

### 2.4. Data Analysis

All responses in section two of the questionnaire were coded as +1 for a correct response, −1 for an incorrect response and 0 for an unsure response. The total score, number of correct responses, number of incorrect responses, and number of unsure responses were calculated for all questions, and for each sub-category. This data was transformed into percentages for analysis.

Descriptive statistics for demographic data and knowledge scores are presented as mean ± standard deviation. Independent *t*-tests were used to determine significant differences in any of the knowledge scores between those coaches providing advice and those not. Repeated measures one-way ANOVA’s were used to determine significant differences in knowledge scores between length of time coaching, age, qualifications, coaches rating of nutritional knowledge, how often coaches read about nutrition, whether coaches had received formal nutrition training, previous playing level, and whether coaches had received advice as a player. The level of significance was set at *p* < 0.05.

## 3. Results

### 3.1. Nutrition Advice

Over half the participants provided advice to their athletes (*n =* 93, 57.1%), while 65 coaches (39.9%) did not provide nutritional advice. All of the coaches imparted advice on fluid (100%), recovery (82.8%), nutrients (40.9%), weight control (28.0%) and supplements (15.1%). The reasons for not giving advice were not confident with level of knowledge (50.0%), not viewing nutrition as important for players (21.7%), no time (19.6%) and someone else provides nutritional support (8.7%). The majority of coaches believed that sports nutrition could help improve athlete performance (98.2%) and help prevent injury (79.1%).

### 3.2. Knowledge Rating

**Figure 1:**
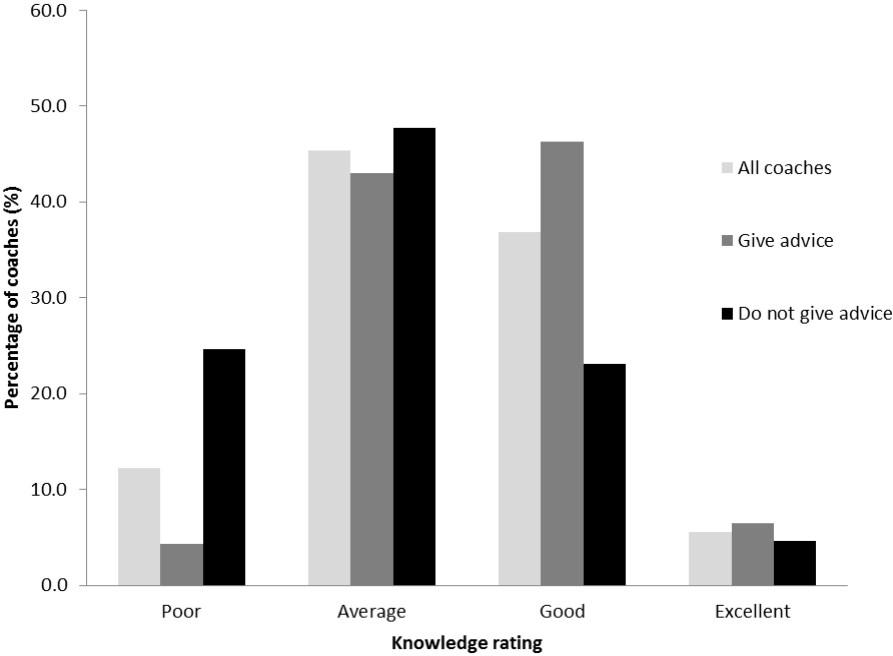
Ratings of coaches own sports nutrition knowledge.

The majority of coaches rated their level of nutrition knowledge as either average (45.4%) or good (36.8%), with 12.3% of coaches rating their knowledge as poor and 5.5% as excellent. Most of the coaches providing advice rated their knowledge as either average (43.0%) or good (46.2%). This contrasted with the coaches that did not provide advice, who had fewer ratings in the good category and more in the poor. Figure 1 illustrates coaches rating of their own sports nutrition knowledge.

### 3.3. Sports Nutrition Knowledge

**Figure 2:**
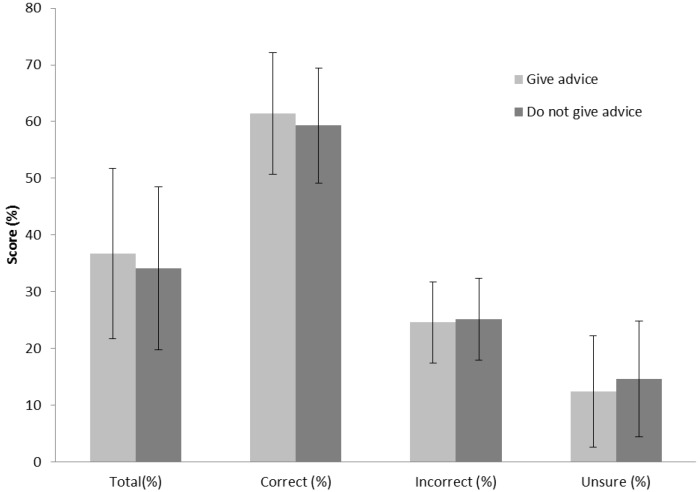
Mean total, correct, incorrect and unsure percentage scores.

The mean total nutritional score for all coaches was 35.4% ± 14.8%. The mean percentage of correct, incorrect and unsure total scores obtained by coaches on the nutrition knowledge questionnaire was 60.3 ± 10.5%, 24.9 ± 7.1%, and 13.4 ± 9.9%, respectively. Figure 2 illustrates the mean total, correct, incorrect and unsure scores obtained by those coaches imparting advice and those not. There were no significant differences in any of these scores between those coaches imparting advice *versus* those who did not (*p* > 0.05).

Table 1 presents the mean total, correct, incorrect and unsure scores obtained by coaches for each nutrition knowledge sub-category. A repeated measures one-way ANOVA demonstrated a significant difference in mean total score between categories for all coaches (*F*(4, 648) = 41.123, *p* < 0.05). *Post hoc* tests identified that coaches achieved significantly higher scores in the nutrients category compared to all other sub-categories, and in the recovery category compared to fluids, weight control and supplements. Following analysis of the mean percentage of correct scores it was found that coaches answered significantly fewer questions correct in the supplements category compared to all other sub-categories.

There was a significant difference in the percentage of correct responses in the fluid category, and the percentage of unsure in the recovery category between those coaches providing advice and those not (*p* < 0.05).

**Table 1:** Sub-category nutritional knowledge scores (*n =* 163).

Sub-Category	Coach Group	Mean Total (%)	Mean Correct (%)	Mean Incorrect (%)	Mean Unsure (%)
Nutrients	All coaches	47.3 ± 16.2	69.3 ± 10.5 ^c^	22.1 ± 8.1	7.9 ± 9.1
	Give advice	48.3 ± 16.1	70.2 ± 10.3	21.9 ±7.9	7.5 ± 8.7
	Do not give advice	46.2 ± 16.7	68.5 ± 10.7	22.3 ± 8.7	8.4 ± 9.7
Fluids	All coaches	19.4 ± 28.6 ^a, b^	47.3 ± 20.5 ^c^	27.9 ± 13.8	0.1 ± 0.2
	Give advice	22.6 ± 30.3	50.5 ± 20.4 ^d^	28.0 ± 14.7	0.1 ± 1.2
	Do not give advice	15.0 ± 26.4	43.1 ± 20.5	28.0 ± 12.9	0.2 ± 1.4
Recovery	All coaches	33.2 ± 31.1 ^a^	62.3 ± 18.3 ^c^	29.1 ± 15.6	4.7 ± 7.2
	Give advice	34.1 ± 32.8	62.7 ± 20.0	28.5 ± 16.2	3.5 ± 6.1 ^d^
	Do not give advice	32.9 ± 28.3	62.2 ± 15.6	29.4 ± 14.7	6.2 ± 8.2
Weight Control	All coaches	25.7 ± 22.5 ^a, b^	57.6 ± 15.1 ^c^	31.8 ± 10.1	7.8 ± 10.2
	Give advice	26.1 ± 23.3	57.3 ± 16.2	31.0 ± 10.0	8.0 ± 10.7
	Do not give advice	26.8 ± 21.3	59.2 ± 13.2	32.4 ± 10.3	7.3 ± 9.7
Supplements	All coaches	18.5 ± 33.6 ^a, b^	38.3 ± 26.4	19.8 ± 17.7	4.6 ± 3.3
	Give advice	21.8 ± 35.1	41.3 ± 26.9	19.5 ± 18.8	4.5 ± 3.4
	Do not give advice	15.0 ± 30.6	34.7 ± 25.0	19.7 ± 16.6	4.8 ± 3.0

Data presented as mean ± SD. ^a^ Significantly less than nutrients; ^b^ significantly less than recovery; ^c^ significantly greater than supplements; ^d^ significantly different from those coaches not providing advice. Statistical significance set at *p* < 0.05.

### 3.4. Nutrition Training

Only 25.2% of coaches had received formal nutrition training, with 73.6% of coaches having received no training in nutrition. Table 2 presents the training nutrition characteristics of those who had received training. Only 31.2% of those coaches providing advice had received formal nutrition training.

**Table 2:** Nutrition training characteristics of coaches (*n =*41).

When (%)		How Long (%)		Form (%)		Update (%)	
Prior to 1999	29.3	Less than 5 h	26.8	Lectures	73.2	Yes	26.8
2000–2004	22.0	Between 5 and 15 h	24.4	Practical Workshop	29.3	No	73.2
2005–2009	34.1	Between 15 and 30 h	14.6	Part of another course	43.9	-	-
2010 onwards	14.6	Longer than 30 h	14.6	Distance Learning	4.9	-	-

### 3.5. Nutrition Information Sources

Coaches were asked to state how often they read about sports nutrition issues. Twenty percent (19.6%) of coaches did not conduct any reading. Other coaches read about sports nutrition on a weekly (8.6%), monthly (35.6%) or six-monthly (26.4%) basis. Coaches who did read about sports nutrition knowledge read the internet (61.1%), followed by journal articles (48.9%), magazines (44.3%), lectures/seminars/courses (26.0%) and sponsors (1.5%). Among the coaches imparting advice to their athletes, 94.6% read about sport nutrition issues.

The majority of coaches (84.0%) did not use an outside professional to provide sports nutrition advice to their athletes. The small percentage that did utilise a professional (14.7%) used a sport nutritionist (66.7%), physiotherapist (50.0%), team trainer (37.5%), personal trainer (25%), registered dietician/nutritionist (16.7%), academic (16.7%) and/or a doctor (8.3%). Among the coaches providing nutritional advice 20.4% used an outside professional.

### 3.6. Relationship between Knowledge Scores and Demographics

No statistically significant differences were found for total knowledge score for all coaches for the variables, time coaching, qualifications, nutrition reading, level of playing experience, and advice received as a player. An independent *t*-test identified no significant differences in total knowledge score between UKCC qualifications (*p* > 0.05). A repeated-measures one-way ANOVA demonstrated significant differences in total knowledge score for age, rating of nutritional knowledge and nutrition training. *Post hoc* tests identified that those participants aged over 51 years achieved a significantly higher score (40.9% ± 16%) than both those in the 18–30 years category (32.6% ± 13.0%) and 31–40 years (31.2% ± 13.4%). Those coaches who rated their knowledge as average achieved significantly lower total scores (31.9% ± 13.7%) than both those who rated their knowledge good (38.9% ± 15.8%) or excellent (47.0% ± 11.2%). *Post hoc* tests for the significant main effect of nutrition training demonstrated that those who had received nutrition training (40.9% ± 17.0%) significantly outperformed those who had not received any nutrition training (33.7% ± 13.5%) on the nutritional knowledge questionnaire.

## 4. Discussion

This study was the first of its kind in the UK to determine the nutritional knowledge of UKCC level 2 and 3 qualified coaches. The main finding demonstrated that the coaches correctly answered 60.3% of the questions; however, due to negative scoring, total mean score achieved was 35.4%. Previous research has set a minimum score of 70% [18] or 75% [1] to determine adequate nutritional knowledge. Although these studies utilised different questionnaires, research [14] which administered the same questionnaire as the current study used this cut off point. Therefore, the results of this study demonstrated that UK coaches have inadequate sports nutrition knowledge. Although previous research has not investigated UK coaches, the results and conclusion are similar to those reported for coaches in North and South America, and New Zealand [1,3,14,15].

Approximately a quarter of the questions were answered incorrectly which substantially reduced the total mean score due to negative scoring. This is of concern as providing incorrect advice is worse than providing no advice [14]. Current research demonstrates that athlete’s nutritional intake is inadequate to support health and performance [1,7,8,9], and that athlete’s lack nutritional knowledge [4,7,10,11,12]. Therefore, it is of high importance that coaches in the UK disseminate factual nutritional information based on adequate knowledge. The current study demonstrated that 57.1% of coaches imparted nutritional advice and there was no significant difference in mean total knowledge score between those coaches providing advice and those not. Similar to the previous point it is of concern that those coaches imparting advice did not have any greater nutritional knowledge.

The coaches answered significantly more questions correctly in both the nutrients and recovery categories compared to fluids, weight control and supplements. These findings are similar to those of another research group [14] who found that New Zealand premier club rugby coaches achieved the greatest score in the nutrients category followed by recovery, fluid, weight control and supplements. However, other authors [1,15] have found that American University coaches scored lowest in macro-and micro-nutrients and highest in supplements. It is difficult to make comparisons with these studies due to the different nutrition questionnaires utilized and the participant sample. Although coaches scored relatively high in the nutrients category (69.3%), only 40.9% of the sample provided advice in this area. Similarly, all coaches provided advice in fluids yet the mean percentage of correct responses was only 47.3%. This is of concern as it may be assumed that coaches are providing advice in an area that they believe they are knowledgeable in when in fact they are inaccurate. Similarly, in an area in which coaches are most knowledgeable they are not providing this advice to their athletes. It is encouraging that only 15.1% of coaches provided advice on supplements as coaches scored significantly lower in this category compared to all others.

The level of UKCC qualification had no impact on nutritional knowledge. Formal coach education is less valued than experiential learning and other less formal opportunities [19,20]. Other researchers have stated that current coach education is not informative or influential [21,22]. Therefore, coach education qualifications do not appear to be sufficiently preparing UK coaches to impart nutritional advice although in both courses there are aspects of sport nutrition learning.

Regarding informal learning, a high percentage (80.4%) of coaches read about sports nutrition issues. However, this had no impact on nutritional knowledge. The majority of coaches used the internet to source their information but this is limited to due to its varied quality. It is encouraging to note that almost half (48.9%) of coaches consulted journal articles. As peer reviewed sources of information, coaches can be confident of obtaining high quality information. However, previous research has demonstrated the existence of a gap between sports science research and coaching practice [23], therefore, there is a need to ensure that sports science research is disseminated in coaching forums and sport specific magazines, and appropriate “lay” language is used [24]. This may aid in the ability of coaches to use academic information and incorporate it into their coaching with confidence.

The one demographic that did distinguish between coaches was those who had received formal nutrition training. These coaches achieved a significantly higher percentage of correct responses than those who had not. This finding may indicate that continued professional development (CPD) is important in developing coach’s knowledge. The coaching system in the UK is not licensed and thus once coaches achieve their UKCC qualification they are not required to undertake CPD to maintain it. Furthermore, only 31% of qualified coaches undertake CPD [25]. This is similar to the current study which demonstrated that only 25.2% of coaches had undertaken formal nutrition training even though the majority recognised the importance of nutrition for performance and injury. Previous research [3], concluding that Brazilian coaches were inadequately prepared to provide nutrition advice, found that only 41% of their sample had undertaken nutrition classes. However, they did not provide any analysis of total score comparisons between the two groups. Therefore, the lack of compulsory CPD for coaches may be having a negative impact on the quality of coaching with regards to all aspects of the coaching process.

The coaching process involves “ologies” (e.g., psychology, biomechanics, exercise physiology, nutrition), sport specific knowledge (technical/tactical) and pedagogy (e.g., motor cognitive learning, coach behaviour) that interact to achieve the end goal of coaching [26]. However, current research has demonstrated that coaches rated their educational needs for advanced topics, such as sports nutrition, significantly lower than both interpersonal coaching skills and physical aspects of coaching [27]. Although this study was conducted with American youth sport coaches, and thus its application to a UK sample is limited, it does provide some evidence that coaches may not undertake CPD in sports nutrition as they do not view it as important to the coaching process. In support of this, the UK Coach Tracking Survey [25] has demonstrated that the knowledge sought by coaches was not related to advanced topics but to sport specific knowledge and the pedagogy of coaching. However, this survey may not have allowed participants to select these types of topics. Therefore, as well as a lack of compulsory CPD it may be that coaches do not fully understand the importance of nutrition in the coaching process.

The majority of coaches did not use an outside professional to provide sports nutrition advice. This may be because of the level of athletes the coaches were working with and thus they did not view it as important, or the coaches did not have access to appropriate professionals. However, this intensifies the point, that if UK coaches are not using outside professionals then it is of greater importance that these coaches have adequate knowledge to impart nutrition advice. The small percentage of coaches that did utilise an outside professional used a variety of sources with the main professional being a sports nutritionist. Currently in the UK official sports nutrition registration is a voluntary process via the Sport and Exercise Nutrition Register (SENr). Therefore, there may be use of unregulated individuals who are not appropriately qualified and experienced to have the competency to work with a range of performance or participation athletes. Similarly, other outside professionals that were used may not be competent to deliver sport and exercise nutrition advice such as physiotherapists, team trainers or personal trainers. Thus it is not only important that coaches have adequate nutrition knowledge but also they understand the industry and who is competent.

There are some limitations to the current study which should be recognised. The current questionnaire failed to identify what level of athletes the coaches worked with. In fact 27.1% of coaches who did not impart nutrition advice cited irrelevant as the reason why. It is assumed that these coaches may have worked within the participation domain. However, nutrition is as important for those participating in physical activity, sport and exercise for health. Therefore, coaches must have adequate nutritional knowledge within all domains of sports coaching. Secondly, gender of the sample was not examined and thus no gender differences could be identified. Previous research [3,15] has reported the demographic of the sample with regards to gender but have not identified if it impacted on nutritional knowledge. Therefore, the fact that this study does not include this information is of minor concern.

## 5. Conclusions

In conclusion, the UK coaches sampled in this study did not possess adequate nutritional knowledge to impart the correct advice to their athletes. Those coaches who had undertaken formal nutrition training achieved significantly higher scores than those who had not. However, this knowledge was still inadequate. Ensuring coaches continue to take part in CPD, including sports nutrition specific courses, is important and it may require regulation to ensure the coaching system is effective. Furthermore, coaches should gain knowledge in a range of disciplines to ensure a holistic approach to coaching, with sports nutrition being integral to this multi-disciplinary philosophy. Although this study is not without its limitations, cautious interpretations should not limit its value as the first of its kind to investigate the knowledge of UKCC qualified coaches. Future research may want to compare knowledge across coaching domains, specific categorised sports (e.g., power; aesthetic), and/or provide qualitative insight into the development of coaches nutritional knowledge.
